# The interaction between the environment and embryo development in assisted reproduction

**DOI:** 10.1590/1984-3143-AR2023-0034

**Published:** 2023-08-28

**Authors:** Urban Besenfelder, Vitezslav Havlicek

**Affiliations:** 1 Department of Biomedical Sciences, Institute of Animal Breeding and Genetics, Vienna, Austria

**Keywords:** assisted reproduction, embryo development, environment, oviduct, endoscopy

## Abstract

It can be assumed that the natural processes of selection and developmental condition in the animal provide the best prerequisites for embryogenesis resulting in pregnancy and subsequent birth of a healthy neonate. In contrast, circumventing the natural selection mechanisms and all developmental conditions in a healthy animal harbors the risk of counteracting, preventing or reducing the formation of embryos or substantially restricting their genesis. Considering these facts, it seems to be obvious that assisted reproductive techniques focusing on early embryonic stages serve an expanded and unselected germ cell pool of oocytes and sperm cells, and include the culture of embryos outside their natural habitat during and after fertilization for manipulation and diagnostic purposes, and for storage. A significant influence on the early embryonic development is seen in the extracorporeal culture of bovine embryos (in vitro) or stress on the animal organism (in vivo). The in vitro production per se and metabolic as well as endocrine changes in the natural environment of embryos represent adequate models and serve for a better understanding. The purpose of this review is to give a brief presentation of recent techniques aimed at focusing more on the complex processes in the Fallopian tube to contrast in vivo and in vitro prerequisites and abnormalities in early embryonic development and serve to identify potential new ways to make the use of ARTs more feasible.

## Introduction

The establishment and use of reproductive techniques represent a potential way beyond natural selection to obtain a higher number of offspring from genetically selected parents for breeding, including for pre-implantation diagnostics. These techniques make an enormous contribution to accelerating breeding progress, allow a better consideration of several complex breeding traits and have thus become a central component of sustainable animal breeding programs (Berglund, 2008; Ferré et al., 2020; Georges et al., 2019). One success that has dominated breeding so far has been the steady increase in milk yield. Meanwhile, however, the high level of milk production with all its metabolic consequences is also held responsible for drastic losses in reproduction (Lucy, 2001; Diskin and Morris, 2008). This development shows that achieved breeding goals inevitably have a negative impact on some traits and thus reveal an antagonism (competition between fertility and production traits) (Roxström et al., 2001; Berglund, 2008).

If assisted reproduction techniques are used for breeding of genetically valuable animals and early embryogenesis is shifted to the laboratory, trait antagonisms affecting fertility and possible environmental influences are opposed to breeding success. It affects precisely those developmental stages that are particularly sensitive to environmental changes, which becomes manifest both, in the short term during embryo development as well as in the long term postpartum through modifications in the phenotype (Duranthon and Chavatte-Palmer, 2018). There is much evidence that these generated embryos are of inferior quality and therefore both ex vivo and in vitro impairments similarly exert influence on these vulnerable stages. In general, more or less all reproductive techniques have been shown to result in the birth of a calf. The gold standard of comprehensive understanding is based on physiological embryogenesis, i.e. the complete development of the gametes in the animal, followed by fertilization and embryo development to succeed in a competent fetal growth and finally the birth of a healthy calf. Development of early embryos appears to be a sensitive indicator for disorders, which becomes evident in a reduction of embryo survival during the first days and weeks after fertilization (Wiltbank et al., 2016; Diskin and Morris, 2008), in long-term consequences (Farin et al., 2006; Duranthon and Chavatte-Palmer, 2018) and finally in a limited suitability of embryo technologies for the application of ARTs (Fèrre et al., 2020).

Among most ARTs, in vitro production aims at culturing oocytes and embryos under artificial conditions in the laboratory for a long time and poses one of the greatest challenges to early embryo development. Interestingly, IVP has resulted in the strongest economic benefit among ARTs in recent years. Where in 2009 the proportion of embryos produced in vitro accounted for half of the embryos obtained via superovulation (Stroud, 2010), in 2018 there were already twice as many and in 2020 three times as many embryos obtained via IVP compared to superovulation (Viana, 2019, 2021). Accordingly, over the past 40 years, much research has been carried out on the IVP, resulting in an extensive literature (Lonergan, 2007). In the meantime, the technology has reached a level that allows, within a very short time, detection and visualisation of a large number of traits and details, scanning micro-structures and below, displaying complex molecular-genetic and metabolic correlations and spectra of effects and to cluster functional areas, which can be assigned to embryo activity. Nevertheless, practical aspects directly related to the application of IVP should also be prioritized as many analytical details in cattle and basic prerequisites still have to be explored. There are still open questions between the scientific knowledge obtained in this field and its implementation for application regarding important key points of environmental factors, plasticity of the embryos during their early development phase and methodological challenges, as the following examples might illustrate:

1. Quality of in vitro-produced embryos is still significantly lower compared to ex vivo (Merton et al., 2003; Ferré et al., 2020; Ealy et al., 2019).
2. Recipient animals do not necessarily have to have contact with the embryos for the first 7 (up to 16) days (Betteridge et al., 1980).
3. On day 3 after insemination, there was no detectable response of the epithelium in the Fallopian tube to the presence of the embryo (Rodríguez-Alonso et al., 2019).
4. Results from heterologous in vivo culture in sheep oviduct resulted in the development of high-quality bovine embryos (Lazzari et al., 2010).
5. The addition of substantial amounts of oviduct fluid to in vitro culture media negatively affects embryo development (Lopera-Vasquez et al., 2017).

In this context, processes in the Fallopian tube have been repeatedly emphasized as the decisive basis and orientation for the needs of early embryonic development (Leese et al., 2001; Ferré et al., 2020; González-Brusi et al., 2020; Saint-Dizier et al., 2020; Dissanayake et al., 2021).

More than 25 years ago, our working group established an endoscopic approach to access the bovine oviduct in order to perform comparative studies in early embryo development in vitro and in vivo (Besenfelder and Brem, 1998). The idea behind this was to identify factors that optimize the feasibility of ART´s in bovine breeding. Therefore, in the following, the anatomical features and the basic tasks of the oviduct as a physiological template for embryonic development are briefly outlined, before moving on to possibilities that show the use of the Fallopian tube in connection with various environmental conditions and embryo growth. Most of the experiments shown below were done by our team or were performed in collaboration.

## Physiological requirements

Reproduction techniques have always been measured against physiological processes in animals. Early embryo development takes place mainly in the oviduct before the embryo reaches the uterus and sends signals to prevent luteolysis. Oocytes and sperm cells enter the Fallopian tube from different directions, meet and fuse via the fertilization process [see Hunter (2008)]. In the Fallopian tube, the embryos undergo further cleavage (Besenfelder et al., 2008), during which some peculiarities take place such as epigenetic tuning (Reik et al., 2001) and timing of major genome activation in the 8 to 16 cell stage (Graf et al., 2014). Mitochondria, with which the oocyte has already been equipped, are adequately distributed to the blastomeres during embryo cleavage and migration phase in the oviduct and first undergo de novo synthesis again in the blastocyst stage (May-Panloup et al., 2005). Transition to the uterus is expected to occur from around day 4 (Croxatto, 2002).

The Fallopian tube is divided into three sections (infundibulum, ampulla, isthmus) which are responsible for embryo nutrition and migration [see Hunter (2012), Yániz et al. (2000)]. The embryo is surrounded by tubal fluid [see Leese (1988); Hunter (2012)], which creates the physical conditions for the processes in the Fallopian tube providing the proper viscosity, pH value and osmolarity (Menezo and Guerin, 1997; Hugentobler et al., 2004). Overall, the oviduct is equipped with secretory and ciliated cells whose ratio and activity are subjected to cyclic changes (Uhrín, 1983). Cells carrying cilia, the circular and longitudinal muscles of the tube ensure cycle-dependent and stage-specific transportation of the embryo along the tubal sections up to the tip of the uterine horn (Ruckebusch and Bayard, 1975; Kölle et al., 2010). The epithelial cells, which have secretory properties, are responsible for the nutritional supply such as carbohydrates, fatty acids, proteins, enzymes and amino acids, and ions (Killian et al., 1989; Menezo and Guerin, 1997; Hugentobler et al., 2007a, b, 2008, 2010; Jordaens et al., 2017). Cytokines and growth factors are thought to have a decisive modulating effect on successful embryogenesis (Neira et al., 2010; Tribulo et al., 2018). Furthermore, microvesicles acting as a carrier for diverse biomolecules (i.e. mRNAs, miRNAs, proteins) are ascribed an important guiding function (Salilew-Wondim et al., 2020).

### Impact of the Zona pellucida

A special feature that characterizes the passage of embryos through the Fallopian tube is their covering, the zona pellucida (ZP), which surrounds and protects them (Van Soom et al., 2010). It seems important to note here that the embryos do not have direct contact with the tubal epithelium during the migration phase through the oviduct due to the ZP that surrounds them. The ZP is an important prerequisite for embryo development, passage and later implantation, from which the embryo in the blastocyst stage hatches after uterotubal passage (Negrón-Pérez and Hansen, 2017).

### Impact of the oviduct fluid

For all processes taking place in the Fallopian tube, the fluid plays a central physical role. All active substances are either in dissolved form in this fluid or are packed in vesicles and are exchanged between the epithelium and the embryo. The fluid facilitates migration of the embryo through the oviduct. Reports on the amount of oviduct fluid vary between species and are significantly influenced by the status of the ovarian cycle. Overall, it can be assumed in cattle that about 1.0 ml of fluid is produced in the oviduct in 24 h during the estrus phase and 0.1 - 0.2 (0.4) ml/24 h of fluid during diestrus (Roberts et al., 1975; Kavanaugh and Killian, 1988; Killian et al., 1989; Kavanaugh et al., 1992; Dickens and Leese, 1994). Based on the surface area (Yániz et al., 2000) and the density of the secretory cells in the individual tubal sections, the ampulla appears to have the greatest secretory capacity. The Fallopian tube is described as a small tube. However, the inner surface area reaches a considerable extent through multi-layered folds and crypts but there is only a capillary gap for the embryos to migrate and to exchange molecules (Kölle et al., 2010; Yániz et al., 2000). The epithelium of the Fallopian tube is covered with a film of fluid to ensure maximum humidity and to fill up the small and narrow capillary spaces.

### Non-physiological environmental conditions

The structure of the Fallopian tube shows that complex processes take place correctly and according to a strict time pattern in this microenvironment. In this context, the diverse approaches in the in vitro production of bovine embryos are to be understood, which make it impossible to follow the entirety of all processes in the oviduct, but which have set priorities for culture imitation in specific biological as well as technical fields:

The aim of using only chemically defined media is to control and ensure proper functioning and to make unbiased statements about changes in the composition of the culture media based on Good Laboratory Practice (GLP) and Good Manufacturing Practice (GMP). In addition, these media are offered commercially and comply with international standards and guidelines that are also required by international societies (Van der Valk et al., 2010). Static monocultures are easier to manage, while medium renewal or use of different media during embryo culture (sequential media) aim to remove metabolites from the embryo environment or to adapt to the changing culture requirements of the cleaving embryos [see Ferré et al. (2020)].

Due to the large number of signaling molecules and metabolic peculiarities for in vitro cultures of embryos, media with more complex additives are applied that can be used via co-cultures (Van der Weijden et al., 2017; Carvalho et al., 2017) or obtained directly from the animal (such as follicular fluid, oviduct fluid, serum) (Van der Valk et al., 2018;). Other, more sophisticated, systems mimic the microtubular anatomy of the Fallopian tube (Beebe et al., 2002). Ferraz et al. (2018) aimed to improve the quality and genetic integrity of IVF embryos by developing an instrumental approach to providing a Fallopian tube on a chip. Finally, Rizos and co-worker transferred the whole oviduct into the petri dish for the culture of bovine embryos (Rizos et al., 2010b).

All in all, the in vitro approaches developed over decades focus on a multitude of elementary peculiarities of early embryo development in vitro. However, combining all these achievements and findings appears to be very complex, extremely costly and hitherto impossible and confirms the uniqueness of the Fallopian tube in early embryo development (Leese et al., 2001). Reproductive techniques, especially the in vitro production of bovine embryos, are still far from optimal at this sensitive stage of development (Sirard, 2017, 2021) and should be further refined to be more efficient and sustainable for use in animal breeding (Ealy et al., 2019). In the following, some in vivo approaches are presented in order to meet demands that directly show the influence of environmental changes on the embryonic stages that are used in assisted reproductive technology (González-Brusi et al., 2020).

## Physiological environment - reflections of environmental disorders in embryo development

Surgical methods have been applied in various ways (Rowson et al., 1969) and have also been successfully used mainly in sheep (Lazzari et al., 2010), which, however, have not been established or maintained in practice to date. Currently, there is a transvaginal endoscopic access to the bovine oviduct, which has been further developed over several years, constantly improved and adapted to various forms of application (Besenfelder et al., 2010). This technique is now easy to use from a technical and anatomical point of view, is minimally invasive and can be performed within a short time. Moreover, the use of transvaginal endoscopy in cattle, which was first presented more than 25 years ago (Besenfelder and Brem, 1998; Besenfelder et al., 2001), is now being applied more and more to determine environmental influences on early embryo development. This technique is thus available for routine applications in practice as well as for processing of scientific questions (Lonergan and Fair, 2008).

### Zona pellucida properties

The nature and properties of the zona pellucida are not only designed for the mechanical stability of the embryo during transport through the Fallopian tube, but also for protection against microorganisms and viruses (Van Soom et al., 2010) and modulate the transzonal exchange of nutrient substrates and messenger substances in the direction of the embryo and back (Clark, 2010; Kölle et al., 2010).

In order to illustrate these transzonal activities, the zonae pellucida of different stages of bovine embryos were obtained endoscopically from the Fallopian tube, morphometrically recorded and compared to in vitro produced embryos. The analysis was performed using scanning electron microscopy. When measuring the pores on the in vivo zonae, it was shown that up to the morula/blastocyst stage, the number and size of the pores became smaller. Compared to the zonae of in vitro cultured embryos, the surfaces of the zonae obtained ex vivo were almost completely covered with secreted granules and the pores were no longer visible. In contrast, 30 to 50% of in vitro embryos showed partial degeneration of the outer zona layer (Mertens et al., 2007). In order to display the zona in its layers, a 10 - 20 µm hole was drilled with a laser. It was found that in in vitro embryos, the outer layer of the zona accounted for 7.5% of the total zona thickness in the zygote and approximately 10% in the later stages. In contrast, the zonae of ex vivo flushed embryos showed a proportion of 18% in zygotes, which increased up to 30% in progressive stages, which was also associated with the disappearance of the reticular structure. This study shows that in vitro and in vivo zonae show significant differences, which are seen as a crucial influence of transzonal exchanges of nutritional and signaling factors in the oviduct (Mertens et al., 2006, 2007).

### Gene expression outlines

Merton et al. (2003) impressively illustrated how the gradual transfer of embryo development from in vivo to the laboratory affects culture results. It was shown how the origin of the oocyte and embryos affects the culture result when embryos are produced either by natural cycle, or by superovulation with/without partial in vitro culture, or by ovum pick-up of slaughterhouse ovaries following in vitro maturation, fertilization and culture. It becomes clear from this presentation that with each successive step performed in vitro, the blastocyst rate decreases significantly (Merton et al., 2003).

In a large-scale study, alternative in vivo and in vitro culture conditions have been examined at the time of fertilisation, major embryonic genome activation and blastocyst formation. Embryos were flushed out of the Fallopian tube at different time points and cultured in vitro up to the blastocyst stage. Vice versa, embryos from in vitro culture were transferred into bovine oviducts at different time points and re-collected from the uterine horn on day 7. Embryos whose development took place exclusively in the animal for 7 days served as a control. From this large-scale study, it was found that changing culture conditions from in vitro to in vivo and vice versa had no effect on embryo development rates (Gad et al., 2012). However, the origin of the oocyte per se had a marked impact on the culture outcome in favor of embryos originating from the oviduct, as also confirmed by other studies (Rizos et al., 2002). The ontological analysis showed mainly contrasting expression patterns in the area of lipid metabolism and oxidative stress between in vivo and in vitro obtained blastocysts. Embryos in the 8-cell stage, around the time of major EGA, were particularly sensitive to cultural environmental change. The study revealed molecular mechanisms and signaling pathways that are especially influenced by in vitro culture (Gad et al., 2012).

It is also known that hormonal stimulation of the ovaries to induce multiple follicles and ovulations also significantly affects the environment in which the embryos develop for the first 7 days. To learn more about the impact of blastocyst development under abnormal endocrine conditions, a study was performed in which heifers were superovulated. On day 2, in half of the animals the oviducts were flushed and these embryos were transferred to heifers having a single ovulation. From both groups, the embryos were flushed on day 7. Here it could be shown that the development up to the morula or blastocyst clearly depends on the endocrine environment. The ratio of recovered blastocysts to morulae was approximately 0.5 in the superovulated heifers, whereas this ratio was 1.8 in the heifers having only one ovulation. These results provide evidence that hormonal use during superovulation negatively impacts embryo cleavage and slows down blastocyst development. Additionally, the embryos were subjected to global gene expression analysis (Bovine Genome GeneChip 100 Format Array). The superovulation treatment triggered higher cellular and metabolic activities in the embryos. Oxidative phosphorylation genes, which are involved in various metabolic, translational and transcriptional processes, were highly expressed in superstimulated heifers compared to the embryos from unstimulated animals (Gad et al., 2011).

### Epigenetic effects

In addition to the different expression signatures in response to environmental effects, these changes in embryos can also be identified using DNA methylation patterns [see Sirard (2021)]. In order to also show epigenetic effects on in vitro/in vivo culture, the DNA methylation pattern of bovine embryos obtained ex vivo was determined in an experimental design before, during and after major embryonic genome activation (EGA). For this purpose, 2-, 8- and 16-cell stage embryos were flushed out of the Fallopian tube and subjected to in vitro culture up to the blastocyst stage and a genome-wide DNA methylation analysis was performed. As expected, not as many blastocysts developed from the flushed 2- and 8-cell embryos compared to the obtained 16-cell stages. These differences were also reflected in the increased number of differentially methylated genomic regions (DMRs) found in blastocysts cultured longer in vitro (from 2- and 8-cell stages) compared to control embryos which developed in vivo. A total of 1623 genomic loci, including imprinted genes, were hypermethylated in blastocysts from all groups (2-, 8- and 16-cell flush), indicating genomic regions sensitive to in vitro culture at each stage of embryo development (Salilew-Wondim et al., 2018).

### Chromosome instability (CIN) in cleavage-stage embryos

There are numerous studies showing that chromosomal aberrations occur more frequently in in vitro produced embryos (Viuff et al., 2002). However, little is known about the comparison of chromosomal stability of in vivo and in vitro embryos that are in the cleavage stage. Therefore, the rate and nature of chromosome instability (CIN) between embryos obtained in vivo and cultured in vitro was examined and compared in a study design. Five Holstein-Friesian heifers were used to isolate single blastomeres from embryos obtained from the same animals (i) ex vivo, (ii) produced in vitro (IVM-IVF), and (iii) from ovarian stimulation with subsequent in vitro production (OPU-IVF).

The individual blastomeres isolated from the embryos were processed for genome amplification and hybridized together with the total DNA of the donor cows (mothers) and the bull (father) on Illumina BovineHD BeadChip arrays. In addition, DNA was analyzed from the parents of the cows (paternal and maternal grandparents respectively) and from the parents of the bull. A genome-wide haplotyping and copy number profiling was then carried out to record the genomic structure of 171 individual bovine blastomeres from the three study groups. The blastomeres from the embryos of both in vitro groups (CIN: 69.2% of the OPU-IVF embryos; 84.6% of the IVM-IVF embryos) showed a strong impairment of the genomic stability. In embryos produced in vitro, the frequency of whole chromosomal or segmental aberrations was significantly higher than in those obtained ex vivo (18.8%). Although the occurrence of CIN was also seen in in vivo embryos, this study illustrates that in vitro production exacerbate chromosomal abnormalities during early embryonic development, thereby significantly impairing the developmental competence and viability of the embryos (Tšuiko et al., 2017).

### Embryo development in heifers and dairy cows

Similarly, dairy cows are subjected to an enormous metabolic stress and, accordingly, loss of weight. It is well accepted that embryo development is very much affected by these environmental conditions and these restrictions are comparable to various IVF procedures (Sirard, 2017). Most importantly, they have a significant impact on postpuerperal fertility, including early embryonic loss (Diskin and Morris, 2008; Wiltbank et al., 2016). A key function is assigned to progesterone, which plays an important role in both folliculogenesis and the establishment and maintenance of pregnancy. As a result of high metabolic activity post partum, high milking dairy cows are not able to provide enough progesterone to support embryo implantation [see Lonergan and Sánchez (2020)]. In order to investigate this limitation and thus the importance of progesterone, embryos generated in vitro were endoscopically transferred into the Fallopian tubes of (i) single-ovulating heifers (controls and progesterone tuned), (ii) nulliparous Holstein heifers and postpartum lactating Holstein cows and (iii) in postpartum Holstein cows (dried-off vs. milking cows) and recovered on day 7 post-oestrus.

The experiments in heifers demonstrated that development to the blastocyst stage is not affected by progesterone administration, but at the molecular level, progesterone-induced changes in the embryonic transcriptome may become manifest later in the post-hatch period (Carter et al., 2010). In the second experiment embryos were transferred into nulliparous Holstein heifers and postpartum Holstein dairy cows which differed in their blood serum progesterone concentrations. In the heifers, 79% of the embryos could be recovered, while cows showed a recovery rate of 57%. Based on the number of transferred embryos, approximately 3 times as many blastocysts could be obtained from the heifers compared to the cows. Of the embryos recovered, 33.9 ± 3.6% had developed to the blastocyst stage in heifers compared to 18.3 ± 7.9% in the post partum cows. There was no evidence of a difference in blastocyst quality as illustrated by the total cell count in the blastocysts (71.2 ± 5.7 vs. 67.0 ± 5.3) (Rizos et al., 2010a). Cows in lactation and cows that were not milked after birth showed significant differences in body weight and metabolic profile. In the first 90 days pp, the cows that were not milked had higher body weights and thus a higher BCS, higher insulin, glucose and IGF1 concentrations in the blood, but lower ß-HBA and NEFA values. The transfer of embryos into the oviducts of these animals resulted in a similar recovery rate, however, the development of the embryos to blastocysts was higher in the non-milked cows at day 7 (39.6 vs. 26.3%) and 8 (49.3 vs. 32.6%) (Maillo et al., 2012).

In summary, the reproductive tract of the postpartum lactating dairy cow is less able to support early embryo development compared to non-lactating heifer and this may contribute to low pregnancy rates observed in such animals. All of these experiments indicate that dairy cows display severely impaired environmental conditions for early embryo development and point to the reason for early embryonic mortality (Rizos et al., 2010a; Maillo et al., 2012).

Finally, it should be pointed out that even asynchrony between the embryo and the recipient limits embryo development. To demonstrate this as well, matured and fertilized oocytes were transferred ipsilaterally into each Fallopian tube of day 1 (n=20) or day 3 (n=20) synchronized recipients. The animals were slaughtered on days 3, 6 or 14 after transfer and the developmental status of the embryos was determined. On days 3 and 6 of slaughter, a greater number of degenerated and retarded embryos was found from asynchronous transfer than from synchronous transfer. On day 14 of slaughter, a clear elongation of the embryos could already be determined. However, only 50% of the asynchronous transfers delivered elongated embryos, whereas all recipients with synchronized ET resulted in conceptuses (Rodríguez-Alonso et al., 2020).

### Cryo-resistance

Post-freezing results also show the impact of the environmental effects and thus the cryoresistance of embryos originating from different culture systems. Thus, Lazzari et al. showed that bovine IVM/IVF embryos cultured in the ovine oviduct prior to cryopreservation were similar to ex vivo embryos and hardly differed from them in terms of the pregnancy rate after transfer (Lazzari et al., 2010). Lonergan et al. (2003) divided the embryo culture into an in vivo and an in vitro culture period. Bovine embryos, which were first cultured for 4 days in the sheep oviduct and then maintained in vitro, did not differ from their in vitro counter partners after thawing. However, embryos that stayed in the oviduct for the last 4 days were significantly better (Lonergan et al., 2003).

Experiments performed with bovine oocytes obtained in vitro and transferred in the bovine oviduct clearly showed that the duration of in vivo culture of embryos is crucial for survival after cryopreservation. To demonstrate this fact, matured oocytes were co-incubated with sperm cells in a glass capillary for 3-4 hours before transfer into oviducts of cattle that had just ovulated. A second set of in vitro embryos was transferred to Fallopian tubes at the 4-8 cell stage. Embryos of both groups were retrieved on day 7 and frozen directly (day 7 embryos) or one day later (day 8 embryos). After thawing, it was clearly shown that the duration of the stay in the Fallopian tube affects cryosurvival. The longer the embryos were in the Fallopian tube, the higher their rate of re-expansion and hatching (Havlicek et al., 2010).

## Conclusion

There is no doubt that embryos can be obtained in vitro and successfully transferred again. The numerous holistic studies carried out to date are seen as a significant contribution to the understanding of fertility and are just as important in the context of assessing fertility problems. From a qualitative point of view, the question still remains open what factors are needed by an embryo and what conditions have to be provided to a conceptus to enable it to develop into a healthy organism in the long term. It is worth mentioning that not only from a scientific point of view the in vivo and vitro development of bovine embryos are still far apart, but also from a practical point of view there is a large gap between these two culture forms. Attempts to close this gap with fluid from the Fallopian tube failed, since the direct use of oviduct fluid in relevant concentrations for IVC does not provide any advantages for embryo culture (Lopera-Vasquez et al., 2017). Tubal fluid constantly collected during the oviduct culture period to be used as a sequential media replacement is not yet available.

To bridge the gap between in vivo and in vitro produced embryos, new ways and more viable approaches should be sought that identify those components from the complex regulated processes in the Fallopian tube [see Ghersevich et al. (2015)] that represent key molecules (metabolites, messenger substances, stimulants) to be efficiently used for ARTs in breeding and research.

The endoscopic approach described here has now been further expanded. It has been used to determine oestrous cycle-dependent alterations of pro-inflammatory factors in the bovine oviductal epithelium post partum in dairy cows (Neubrand et al., 2021; Pothmann et al., 2022), for the intratubal insemination of sperm from various treatments (Radefeld et al., 2018), as well as the collection of fluid from the oviduct for the determination of stage-specific tubal components (Pothmann et al., 2017; Papp et al., 2019; Havlicek et al., 2022; Neubrand et al., 2022).
